# Rehabilitation using virtual gaming for Hospital and hOMe-Based training for the Upper limb in acute and subacute Stroke (RHOMBUS II): a qualitative analysis of participants’ experience

**DOI:** 10.1136/bmjopen-2026-119145

**Published:** 2026-07-02

**Authors:** Tom Butcher, Alyson Warland, Victoria Stewart, Meriel Norris, Basaam Aweid, Arul Samiyappan, Elmar Kal, Jennifer Ryan, Dimitrios A Athanasiou, Karen Baker, Guillem Singla-Buxarrais, Carole Pound, Francesca Gowing, Cherry Kilbride

**Affiliations:** 1 Department of Sport, Exercise and Rehabilitation, Northumbria University, Newcastle upon Tyne, UK; 2 Department of Health Sciences, Institute of Health, Medicine and Environment, Brunel University of London, Uxbridge, UK; 3 Alderbourne Unit, Hillingdon Hospital, Uxbridge, Middlesex, UK; 4 Stroke Unit, Hillingdon Hospitals NHS Foundation Trust, Uxbridge, UK; 5 Early Supported Discharge (Stroke), Central and North West London NHS Foundation Trust, London, UK; 6 Adult Services, Central and North West London NHS Foundation Trust, London, UK; 7 School of Physiotherapy, Royal College of Surgeons in Ireland, Dublin, Ireland; 8 Neurofenix, London, UK; 9 Neurofenix, Atlanta, Georgia, USA; 10 Independent Researcher, London, UK

**Keywords:** REHABILITATION MEDICINE, Digital Technology, Stroke, Physical Therapy Modalities, QUALITATIVE RESEARCH, Virtual Reality

## Abstract

**Objectives:**

To explore the experience of participants using the Neurofenix (Naples, Florida, USA) platform for hospital and home-based upper limb rehabilitation in acute and subacute stroke, and to report acceptability of trial and data collection processes in a feasibility randomised controlled trial (Rehabilitation using virtual gaming for Hospital and hOMe-Based therapy for the Upper limb in acute and subacute Stroke (RHOMBUS II)). The trial investigated safety, feasibility and acceptability of the platform, a non-immersive virtual reality (VR) tool, to facilitate upper limb rehabilitation following stroke. The intervention group (n=16) received usual care plus the platform for 7 weeks. The control group (n=8) received usual care only.

**Design:**

A qualitative descriptive approach using semi-structured interviews. Interviews were audio-recorded, transcribed verbatim, and analysed using the Framework Method. The Theoretical Framework of Acceptability informed the development of the topic guide and the analysis.

**Setting:**

Homes of participants and an inpatient stroke rehabilitation unit, London, UK.

**Participants:**

A purposive sample of 11 adults (≥18 years) was recruited from the trial intervention group, 35–89 years of age, ranging from mild to severe upper limb impairment and representing varying levels of engagement with the Neurofenix platform. Additionally three participants were recruited from the trial control group.

**Results:**

Six themes were developed: (1) trial enrolment, indicated that participants enrolled in the study due to hope and for distraction; (2) affective attitude, described feelings associated with the platform, such as the subthemes ‘enjoyment of the games’ but also ‘frustration’, often caused by Wi-Fi connection issues; (3) barriers and facilitators, explored environmental factors such as having a suitable space to use the platform, usability factors such as comfort and the need for support from others; (4) perceived effectiveness of the intervention, described an overall belief that the platform had beneficial effects on both upper limb and cognitive function; (5) self-efficacy, showed how confidence with the platform grew with time and the value placed on performance tracking and routine; (6) trial processes, indicated that assessment burden was manageable and potential allocation to the control group was not a deterrent to trial enrolment.

**Conclusion:**

This study demonstrated the overall acceptability of the platform and offers valuable insights to guide the further development of rehabilitation technologies. This study also adds to the limited literature on the user experience of VR technologies early post-stroke.

**Trial registration number:**

ISRCTN11440079.

**STRENGTHS AND LIMITATIONS OF THIS STUDY**

- Acute and subacute stroke survivors were recruited, allowing the use of rehabilitation technology to be explored across the stroke pathway in both the hospital and home setting.
- Purposive sampling was used to ensure the 11 participants from the intervention group represented a range of ages, severity of upper limb impairment and amount of device use.
- Participants were interviewed by a member of the research team that they had interacted with during the trial which may have impacted the responses of the participants.
- The range of experiences expressed is limited by the sample being across a single stroke pathway.

## Introduction

 Stroke represents the third leading cause of disability worldwide, with global stroke incidence having increased by 70% since 1990.^1^ One in four people will have a stroke in their lifetime, meaning more people are living with the effects of stroke, with this number estimated at 94 million people worldwide.^2^ Approximately 65% of all individuals post stroke, experience difficulty using their affected upper limb for activities of daily living,^3^ resulting in a potential 61 million people living with stroke-related upper limb activity limitations.

It is known that high-intensity, repetitive task practice is a key driver and recommendation for recovery of the upper limb post stroke.^4^ Recovery can be further enhanced by starting rehabilitation early, especially within the first month post stroke.^5 6^ However, this is rarely achieved with the literature suggesting that both the dosage and timeliness of current provision are markedly insufficient to provide optimal benefit to the individual.^7^,^9^

Contributors to these low levels of upper limb rehabilitation can be broadly categorised as relating to the healthcare system or the individual. System-related reasons include limited staff to deliver necessary therapy intensity^10^ and pressure on early discharge driving focus towards the lower limb.^11^ Individual barriers to upper limb stroke rehabilitation include low motivation, lack of self-efficacy and perceived insufficient movement for meaningful practice.^12 13^ Stroke survivors must therefore be involved in the development of potential solutions to ensure they are meaningful, acceptable and feasible, and research using those solutions must explore the experiences of stroke survivors engaging in the intervention.

Rehabilitation technologies are continuously evolving and offer the potential to increase therapy dosage and intensity without significantly increasing burden on staff.^14 15^ For example, virtual reality (VR) rehabilitation platforms can offer a gamified and motivating approach and have been shown to be an effective method of increasing the intensity of upper limb rehabilitation post stroke.^16^,^18^ The Neurofenix (Naples, Florida, USA) platform (www.neurofenix.com, to be referred to as ‘the VR platform’ throughout) is one such example that has been co-developed by stroke survivors, clinical researchers and bioengineers. The VR platform uses NeuroBall, a portable, upper limb controlled therapy device, which translates hand and arm movements into gameplay, displayed on a tablet computer via a uniquely designed rehabilitation gaming app. The development of the VR platform was guided by the Medical Research Council Complex Interventions Framework,^19 20^ progressing from a proof-of-concept study^21^ to a feasibility intervention study,^22^ and most recently a feasibility randomised controlled trial (RCT).^23^ The acceptability of the VR platform has also been demonstrated for home-based use by chronic stroke survivors,^24^ adding to the established body of research exploring home-based upper limb rehabilitation technologies.^25 26^ This paper builds on previous work, by describing the acceptability of the VR platform in acute and subacute stroke within the context of a feasibility RCT,^23^ in which participants in the intervention group (n=16) received usual care plus the platform for 7 weeks and those in the control group (n=8) received usual care only. Additionally, it offers insight into the less-well researched area of the use of rehabilitation technology across the acute and subacute stroke pathway in both the hospital and home setting. The aims of this arm of the study were to explore the acceptability of the VR platform to people with stroke and to explore factors impacting adoption of the intervention. A further aim was to explore the acceptability of trial processes, including allocation to the control group. Staff who provided support were also interviewed about their experiences and these results will be published in a separate paper to inform future trial design and rehabilitation technology implementation.

## Methods

This qualitative descriptive study was embedded in a feasibility RCT with a parallel process evaluation, full details of which can be found in the published protocol^27^ and quantitative results paper.^23^ Qualitative descriptive studies are not based on a specific methodological approach, staying close to the surface of the data as described by participants.^28^ This feasibility RCT followed the local stroke pathway under one stroke consultant (BA) and was conducted on the Acute Stroke Unit (ASU) at Hillingdon Hospitals’ National Health Service (NHS) Foundation Trust (London, UK) and in the homes of participant stroke survivors under the care of the Central and North West London NHS Foundation Trust Early Supported Stroke Discharge (ESSD) team. The intervention consisted of self-directed use of the VR platform over a 7-week period as an adjunct to usual scheduled therapy sessions. Each participant received their own personal Neuroball and tablet computer pre-loaded with the Neurofenix app and were advised to slowly increase their use of the device with the target of reaching or surpassing 45 min use per day. Support was provided to participants through initial training on the platform, delivered by a member of the research team (VS), with additional training/assistance available from VS throughout the 7 weeks when required. Members of staff from the ASU and ESSD teams were also offered training and access to training material on the VR platform to help them support participants. Semi-structured interviews were used to collect data. Fourteen out of the 16 participants in the intervention group were invited to complete interviews using purposive sampling to ensure participants represented diversity in age, severity of impairment and amount of device use (ascertained using data collected from the VR platform). Four out of the eight participants in the control group were also invited to take part to explore their trial experiences and feelings on group allocation.

Separate interview guides were developed for the intervention and control group using the Theoretical Framework of Acceptability (TFA^29^; table 1) and were piloted before use. A copy of the interview guides is included in online supplemental material 1. Interviews were conducted within 2 weeks of either the end of the intervention period (intervention group) or final outcome measure collection (control group) at the homes of the participants or within a private room in an inpatient rehabilitation unit. All participants provided informed consent, and the interviews were conducted by a single member of the research team (VS, a highly experienced neuro-physiotherapist). Acknowledging the professional background of VS as a neuro-physiotherapist with minimal prior experience of rehabilitation technology alongside some initial reservations about its usefulness, a reflexive diary was maintained throughout to examine these potential influencing positional factors, thereby enhancing trustworthiness. All interviews were audio-recorded and professionally transcribed verbatim. The transcripts were anonymised prior to analysis.

**Table 1:** Component constructs of the Theoretical Frame of Acceptability^29^

Component construct	Description
Affective attitude	How an individual feels about the intervention
Burden	The perceived amount of effort that is required to participate in the intervention
Ethicality	The extent to which the intervention has good fit with an individual’s value system
Intervention coherence	The extent to which the participant understands the intervention and how it works
Opportunity costs	The extent to which benefits, profits or values must be given up to engage in the intervention
Perceived effectiveness	The extent to which the intervention is perceived as likely to achieve its purpose
Self-efficacy	The participant’s confidence that they can perform the behaviour(s) required to participate in the intervention

### Patient and public involvement

Members of two community stroke groups provided guidance on the study design and trial documentation, including the participant information sheets, consent forms and interview topic guide. Two additional stroke survivors were on the trial steering committee and were reimbursed for their time and lived expertise. The six principles of the UK Standards for Public Involvement were followed throughout.^30^

### Data analysis

Transcripts were analysed using the Framework Method, which allowed for a hybrid deductive and inductive approach to be taken.^31^ Deductive analysis was conducted using the pre-existing constructs of the TFA (table 1); however, to ensure all relevant and unexpected data was captured, inductive open-coding was used in parallel.^31^ The lead analyst (TB), a neuro-physiotherapist with extensive clinical and research experience of rehabilitation technology and previous experience of qualitative analysis, first read and re-read all transcripts for familiarisation with the data. Three transcripts were then independently coded by TB and a highly experienced qualitative researcher (CK). Initial coding from both the researchers was compared and final codes were then developed by TB into a working analytic framework to be iteratively applied to the remaining transcripts. Minor adjustments to codes were made and some further codes were added until no new codes were identified, resulting in the final framework of 65 codes. Trustworthiness in the analytic process was enhanced through the initial independent dual analysis and coding undertaken by both TB and CK and also through negative case analysis.

Summarised data were charted into a framework matrix using Microsoft Excel. Narrative summaries were composed, and exemplar quotations were identified for all categories. Sub-themes and themes were then developed from the matrix by reviewing the data and connecting related ideas and concepts.

## Results

Fourteen participants (11 intervention, 3 control) completed the interviews. Three intervention and one control participant declined to take part. The 11 participants (3 women) from the intervention group had a mean age of 63 years (range: 35–89 years), a mean Fugl-Meyer Assessment-Upper Extremity (FMA-UE) score of 44 (range: 12–66) representing mild to severe upper limb impairment as defined by Woytowicz *et al*,^32^ and their mean amount of weekly device use was 121 min (range: 35–301 min). The three participants (2 women) from the control group had a mean age of 62 years (range: 52–75 years) and a mean FMA-UE score of 42 (range:0–64), representing mild to severe upper limb impairment. Participant characteristics are summarised in table 2. The average interview time for intervention group participants was 41 min (range 28–75 min), and for control group participants was 24 min (range 13–31 min).

**Table 2:** Interview participant characteristics

Participant number	Group allocation	Age range (years)	Sex	Simplified modified Rankin score*	Fugl-Meyer assessment upper extremity†	Mean (range) weekly device use (min)
1	Intervention	50–59	Male	3	53	80 (14–326)
5	Control	50–59	Female	5	0	N/A
6	Intervention	40–49	Female	5	50	223 (17–587)
9	Control	50–59	Male	2	63	N/A
11	Intervention	70–79	Male	3	55	98 (44–196)
12	Intervention	60–69	Female	1	54	35 (16–72)
14	Intervention	60–69	Male	3	12	111 (70–169)
15	Intervention	70–79	Male	4	15	96 (58–154)
16	Intervention	30–39	Male	2	65	126 (27–206)
17	Intervention	60–69	Male	2	66	103 (56–133)
18	Intervention	80–89	Male	3	63	53 (0–171)
22	Control	70–79	Female	1	64	N/A
23	Intervention	50–59	Male	3	25	301 (136–347)
24	Intervention	80–89	Female	2	28	101 (90-112)‡

* Score of 2=slight disability, 3=moderate disability, 4=moderately-severe disability, 5=severe disability^46^

† Score of 0–15=severe impairment, 16–34=severe-moderate impairment, 35–53=moderate mild impairment, 54–66=mild impairment^32^

‡ Device use for first 2 weeks prior to withdrawal from study

N/A, not applicable.

### Summary of themes

Six themes were developed from the data, summarised in table 3. Exemplar participant quotes are provided with contextual details of sex and severity of upper limb impairment according to their FMA-UE score. Themes 1 (trial enrolment – influencing factors) and 6 (trial processes) contain information gathered from all 14 interview participants and relate to trial-related matters, whereas themes 2 (affective attitude – feelings associated with the platform), 3 (barriers and facilitators), 4 (perceived effectiveness of the intervention) and 5 (self-efficacy) are specific to the 11 participants from the intervention group and align with component constructs of the TFA.

**Table 3:** Themes and subthemes

Themes	Subthemes
1. Trial enrolment – influencing factors	1.1 Distraction 1.2 Hope for own recovery 1.3 Altruistic hope to help others 1.4 Feelings about randomisation
2. Affective attitude – feelings associated with the platform	2.1 Enjoyment of the games 2.2 Game dislikes 2.3 Frustration 2.4 Positive associations and ease of use
3. Barriers and facilitators	3.1 Environmental barriers and facilitators 3.2 Usability of the platform 3.3 Comfort and adverse events 3.4 Support from others
4. Perceived effectiveness of the intervention	4.1 Improvements in upper limb function 4.2 Cognitive engagement 4.3 Perceptions of how the platform helped them 4.4 Recommendations to improve effectiveness
5. Self-efficacy	5.1 Confidence with the platform 5.2 Performance tracking 5.3 Routine and flow
6. Trial processes	6.1 Assessment burden 6.2 Pre-trial information and initial training effectiveness

#### Theme 1: trial enrolment – influencing factors

Understanding why participants chose to take part in this feasibility RCT is important as it may help to inform recruitment processes for later studies, particularly given the current lack of technology trials involving acute and subacute stroke survivors. Ten out of the 14 interview participants stated that they enrolled in the trial in the hope it would help with their recovery. All the participants in the study were recruited to the trial less than 3 weeks after their stroke and were looking for any opportunity that might help them regain movement and function in their impaired upper limb.

Primarily I done it for selfish reasons for myself. To get myself better, you know anything that might help. (P1, male, moderate-mild impairment)

I try anything that, if I think it might improve my ability to move and get back to a normal life. (P14, male, severe impairment)

Throughout the interviews the impact of experiencing a stroke and the aftermath of it on their mental health was mentioned by many participants. This also contributed to trial enrolment as taking part in the study was seen as a positive distraction from such a challenging situation. It also represented an opportunity to take back some control in a situation where individuals often feel this has been lost.

It was a breath of fresh air. It was a very positive experience for me because up until then it was all doom and gloom. It gave me something else to think about other than how bad I was and how I couldn’t do anything. And I felt like I’d got a bit of control back. (Becomes upset). (P12, female, mild impairment)

Five participants, including all three from the control group, expressed a hope that by taking part in the study they would be helping future stroke survivors.

Well this could possibly help somebody. Not only me but help somebody else with a stroke that could be far worse than me. (P11, male, mild impairment)

Regarding acceptability of group allocation, eight participants discussed their feelings about the potential of not receiving the intervention if allocated to the control group. Of the three control group participants, P5 expressed initial disappointment, but accepted it as being part of the process, whereas P22 was pleased to be allocated to the control group. P9 felt indifferent to his allocation.

To be perfectly honest, I was pleased that I didn’t at that time take on something else because I felt that I was … my carers, not the carers, but the people from the ESSD were coming twice a day, … and at that time I probably was feeling more fatigued. That was enough for me to cope with, so, if you like, I was pleased. (P22, female, mild impairment)

Participants in the intervention group expressed relief with their allocation, with the exception of P24, who initially wanted to be allocated to the control group; however, this was because she had not fully understood what the intervention was and once she started using the VR platform was happy with her allocation.

#### Theme 2: affective attitude – feelings associated with the VR platform

Within the TFA, affective attitude describes the feelings an individual has about the intervention. Participants expressed a broad range of emotional experiences associated with using the VR platform. Positive feelings included enjoyment of the games, in particular enjoyment of the competition with a desire to ‘beat the game’.

I needed for me to be able to beat the machine if you like, rather than the machine beat me. So, as far as I was concerned, I needed to do it to know that I could do it. (P18, male, mild impairment)

Game-specific preferences were varied with sometimes contradicting views, illustrating how personal game preference is. Game simplicity was viewed positively by some participants, who saw the games as ‘a bit of fun’ and ‘amusing’, whereas others felt the games were too simple, expressing a desire that the games were more cognitively challenging.

I kind of found the Holiday Dash one was the one that I liked the best […]. I don’t know really, I just found it a little bit of fun trying to get all the things to go on holiday with. (P12, female, mild impairment)

Yeah because you engage your brain, you know. It is not just the physical, […] but mentally you’ve got to train your brain as well and there wasn’t enough of that on there. (P1, male, moderate-mild impairment)

Five participants stated that their experience would have been improved if the platform had more games.

Well the thing is, first and foremost, there’s not enough games on it. (P18, male, mild impairment)

Eight participants reported feeling frustration at some point during the trial. Connection issues to the Wi-Fi were the cause of frustration for six participants, though for two of those participants a clear reason was found as aeroplane mode had accidentally been activated on the tablet.

There were times I wanted to put it out of the window. […]. I couldn’t get it working but the Internet is something went wrong. I didn’t know what was going on […]. So I wanted to use it and I couldn’t. (P6, female, moderate-mild impairment)

Losing to the game was also identified as a cause of frustration, as was difficulty using the platform due to the impact of the stroke.

[Talking about the Space Invaders game] It was so frustrating the way I kept getting killed. (P1, male, moderate-mild impairment)

Again very frustrated because it is something I would have done quite easily before the stroke but that’s all part of it. (P11, male, mild impairment)

Four participants found that as their arm function improved over the 7-week intervention period their relationship with the VR platform changed. One reason given for this was that the games no longer provided sufficient challenge, suggesting the importance of games that can adapt and provide a range of difficulty as the user progresses. The second reason was that their improved arm function allowed participants to engage in more functional tasks again, leaving less time to use the platform.

But I think as the study went on, the problem was that I was starting to be able to do stuff that I hadn’t been able to do for a long time, like once I’d got downstairs I could work the washing machine and dishwasher and everything else and this sort of started to overtake you know. (P12, female, mild impairment)

#### Theme 3: barriers and facilitators

One sub-theme identified was environmental barriers and facilitators, including access to plug sockets to charge the devices, Wi-Fi connectivity, somewhere comfortable to sit and use the platform and noise/distractions. Two participants described the challenges of using the platform in the ward setting, and how much easier it was when they had their own personal space in either the stroke rehabilitation unit or at home.

[Referring to being in the hospital ward] Yes, you know it was so open there nothing was private, yes. And obviously when you’re playing by yourself on your own, you laugh and shout and scream at the ball and you know – you’d probably be a lot more reserved in a ward when there’s all these people around you. And the different times of day. I couldn’t sleep some nights, so I’d do it at 2 o’clock in the morning. (P1, male, moderate-mild impairment)

Aspects of device usability were a potential barrier with 9 of the 11 participants reporting some initial difficulty with donning the Neuroball due to the fitting of the wrist strap and getting the fingers in the correct position, with stretching of the fingers sometimes needed prior to device use. However, participants reported improvements in their ability to don the device over time, with most achieving independence with this between 2 days and 3 weeks.

First of all, I needed assistance putting the ball on my hand every time but now I can do it very easily by myself. [Asked how long this took] Probably about two or 3 weeks. (P14, male, severe impairment)

For some, device comfort was a barrier to use with six participants experiencing discomfort from using the device. For four participants, this was muscle-related due to prolonged use of the device in a single session and was short-lived.

I’ll say cramp, cramp isn’t the right word but it got fixed in that position around the NeuroBall. […] any pain that I experienced was momentary. (P17, male, mild impairment)

For two participants, the discomfort came from contact points between the Neuroball and the hand, though again, this was after prolonged single session use. One participant had to withdraw from the study as she experienced a flare-up of previous carpal tunnel syndrome, which she felt was partly caused by using the Neuroball and partly from the other exercises they were doing.

I think along with all my other exercises, it just generally didn’t help; it didn’t help but not for 1 min thinking it caused it. I just think that it went along with all the other exercises and things to aggravate it, yes. (P24, female, severe-moderate impairment)

For six participants, support from others facilitated their use of the platform. Level of upper limb impairment according to FMA-UE scores did not play a significant role in support requirements though, as two of the most severely impaired participants required support for only a day or two or no support at all. Whereas several participants with mild impairment needed regular support from staff or relatives. Support provided was often physical support with device use, but one participant described how the enthusiasm and interest of one member of nursing staff helped with her own motivation:

One of the nurses was really on board with it and that really helped because she came along, got the ball out for me and I kind of needed that. I think it is important actually if you’ve got like a designated nurse that is really into it. Because she was great, she came along, took the whole thing out and got me motivated to do it. Which was great. (P12, female, mild impairment)

Two participants expressed feelings of not wanting to ask for support, one was due to not wanting to interrupt busy nursing staff, another did not want to ask their family for support as they did not want it to detract from valuable time together.

[Asked about whether family helped with using the platform] No, because the time is so precious with that hour, that is the quickest hour in a day when I got my family here. (P14, male, severe impairment)

#### Theme 4: perceived effectiveness of the intervention

Of the 11 participants in the intervention group, 10 reported that they thought the VR platform had beneficial effects on their upper limb. The remaining participant, who had an exacerbation of a previous carpal tunnel condition resulting in withdrawal from the trial after 2 weeks, reported she was unsure if it had or not. Positive effects reported included reduced pain and improved flexibility.

Yes, definitely, it helped. Definitely after having used it, in those hours after, I definitely would have more range of movement, not so much pain and feel more flexible. (P1, male, moderate-mild impairment)

Additional reported benefits noted were improvements in hand-eye coordination, grip strength and hand control, all of which contributed to recovery of movement and function of the upper limb.

I started to realise that I could actually squeeze it. So it must have helped me build up the strength to do that and that turned out to be one of my favourite things to do on it really […]. I found that very useful and I think it definitely helped me with gripping things. If you consider I couldn’t even squeeze it […], I think that did make a huge difference. (P12, female, mild impairment)

Beyond improvements to the upper limb, seven participants also described beneficial cognitive effects from the intervention due to the concentration required to use the device.

I think it helped the brain make it, making it work. That’s all it’s doing really is making it do something that, again the brain is getting better. (P11, male, mild impairment)

Participants also offered insights into the mechanism underpinning how the VR platform had helped them, with the most common reported reason being that it encouraged them to complete a higher number of repetitions of arm movement than they would have otherwise completed.

It’s a sort of interactive way of actually doing exercises because it can be quite tedious and boring. So if you’re focusing on something and then you’re sort of enjoying it, playing the games, it helps with doing the reps and doing the exercises, yes. (P16, male, mild impairment)

Additional reasons given were that participants felt using the VR platform made them focus more on their affected arm when they could have easily neglected it, and that the haptic vibrations were an effective way of stimulating the hand.

It forced me to sort of use my left hand and left arm. I think that’s the main impact, it just sort of, yeah, not forced, yes almost forcing me cos I had to sort of focus more on the left side a bit more otherwise I wouldn’t, you know I wouldn’t be doing it as much I think. (P16, male, mild impairment)

A range of suggestions were made for how the platform could be further improved. The desire for a wider range of game selection, including cognitive puzzle games, and a higher level of difficulty was raised again. Greater sensitivity of the grasp function to allow training of specific digits was a suggestion made by two participants. Two participants commented on their hand being either too small or big for the device, so their suggestion was for different sizes to accommodate hand size.

#### Theme 5: self-efficacy

There was lower initial confidence with the platform for those with less previous experience of technology and gaming, and higher confidence for those with more experience. Confidence grew with time spent using the platform.

And in like mastering the games and navigating the whole, you know everything obviously your confidence builds, you’re familiar with it. So it got a lot better. A lot easier. (P1, male, moderate-mild impairment)

Routine emerged as being key to regular engagement with the platform. However, what routine meant to participants varied, for example, for P1, it was having an order in which he played the games each time he used the platform; for P17, it was using the platform at the same time each day; and for P23, it was always listening to music while he trained with the device.

Okay, well I tried to do it every afternoon, I had a window of opportunity after lunch and before 4 o'clock, 5 o'clock, when I thought, well I must do the [Neuro] ball and do the exercises and yes, I do them fairly consistently. (P17, male, mild impairment)

All participants reported that performance monitoring features in the app helped to motivate them by allowing them to track their progress and set goals.

I mean when you first log in, I think, it tells you how many days you’ve done in a row but then telling you how many movements you’ve done as well as the time I think. It’s good to be told what you’ve done but also what you’ve got done left if you are going to set yourself a time. But I quite like watching it when the green little circle went round to tell me how many minutes I’d done. (P23, male, severe-moderate impairment)

Some participants were not interested in comparing themselves to others with the leaderboard, preferring to track their own progress to reach a personal goal, whereas other participants were driven to try and get to the top.

It was really good, it gave me the incentive to get to the top. (P1, male, moderate-mild impairment)

#### Theme 6: trial processes

One potential source of burden for participants was the assessment process. All 14 interview participants were asked about their experience of outcome measure collection. While most felt the assessment burden was acceptable, concerns were raised regarding the outcome measures being labourious, requiring a lot of concentration and sometimes being difficult to understand (reported by one participant each).

I wondered what I was doing. I wondered why I was doing the picking the ball-bearing up and putting it on the dot and then picking it up and putting it back on the bottom. I have no idea what that was all about but obviously you know. (P11, male, mild impairment)

Some difficulty using numerical rating scales was also reported by one participant. The remaining participants had no concerns about the assessment process, and two participants expressed positive feelings about the process as it demonstrated their improvement in a clear to understand quantitative metric:

I mean the process again, it was just good to know just to have like a, like a quantitative score to see how I’ve improved […]. So, actually, it was actually quite informative I think, yes. (P16, male, mild impairment)

Overall, participants in both the intervention and control groups felt like they were sufficiently informed about what the trial would involve prior to enrolment; however, two participants from the intervention group commented that they did not really understand the intervention until they received the training on it. One suggestion for future trials was to have a video of someone/the method of using the platform to show potential participants as part of the recruitment process.

## Discussion

These qualitative findings provide varied insights into the experiences of individuals with acute and subacute stroke engaging in VR facilitated upper limb rehabilitation alongside their conventional rehabilitation, within the context of a feasibility RCT. As this study followed the stroke pathway from hospital to home, the findings also contribute to the research gap identified by Cai *et al*^33^ in their recent meta-synthesis of qualitative studies, in which they suggest the need for research exploring how VR stroke rehabilitation can help to create a continuum of care as stroke survivors transition from hospital to the community or home.

As this qualitative study formed part of a feasibility RCT, it was important to learn from participants about their experiences of trial-related matters to inform the development of future trials. Theme 1 explored factors that influenced trial enrolment, with hope that it would help their own recovery being the most stated reason along with a desire to help others by contributing to research. Carlstedt *et al*^34^ also found these to be two of the most common reasons for taking part in rehabilitation research post stroke. The desire to contribute to research may have been a factor in why potential allocation to the control group did not appear to negatively impact trial enrolment, as participants understood they were still contributing regardless of group allocation. An interesting sub-theme that emerged was that taking part in the study provided a form of distraction and an opportunity to take back some control. Distraction both by participation in research and the use of VR, due to its ability to engage and immerse users in the task and virtual environment,^35^ may be of additional value to individuals with subacute stroke as the onset of post stroke depression is most prevalent in the first 3 months.^36^ Providing a sense of taking back control also has potential long-term benefits, as higher internal locus of control has been associated with higher health-related quality of life at 12 months and 18 months post stroke.^37^

Enjoyment of the Neurofenix platform has already been demonstrated in chronic stroke,^24^ and findings from this study have shown the same effect in acute and subacute stroke. As has been consistently demonstrated in VR-based upper limb rehabilitation, the fun of playing the games was a key contributor to enjoyment.^2438^,^40^ The novelty and enjoyment of the games provided did appear to deteriorate with time, however, with the most common game-related complaint being that there were not enough of them to maintain attention and ongoing engagement. Ding *et al*^41^ identified game personalisation as an important influence in patient engagement with VR rehabilitation, which is supported by the varied insights offered by our participants, with some enjoying the simplicity of the games while others found the games too simple to provide sufficient challenge. An example of this was the request for games which address both physical and cognitive rehabilitation needs by incorporating cognitive challenge as well as upper limb movement. This highlights the importance of game development in VR-based interventions becoming long-term management tools, to ensure users have access to a wide range of games which offer varying levels of both physical and cognitive challenge.

All 10 of the participants who completed the intervention period believed that using the VR platform led to improvements in their upper limb. These included greater flexibility, strength and control and reduced pain, all of which improved movement and function. Seven participants also perceived that the platform had aided their cognitive recovery. In keeping with feasibility studies, this trial was not designed to examine effectiveness of the intervention. However, the patient-reported findings concur with the perceived benefits of the VR platform experienced by individuals with chronic stroke^24^ as well as in the literature more broadly.^41^

Connection problems negatively impacted the engagement of participants with the VR platform and were a source of frustration. For those who accidentally activated aeroplane mode on the tablet without realising, this identifies unfamiliarity with technology as being a barrier. This could be easily addressed through further training or instructional resources. However, for those using the VR platform in the acute inpatient setting, connection issues were largely due to poor NHS Wi-Fi connection. This significant organisational and institutional barrier is well known^42 43^ and needs consideration for future trials and for successful implementation of rehabilitation technologies within NHS settings.

As this study investigated the use of the VR platform across the stroke pathway, barriers and facilitators emerged for both in-hospital and at-home use. The lack of private space on the ward detracted from the convenience and flexibility that home-based training offers.^25^ As has been previously reported, those who trained at home benefited from the flexibility of being able to leave the platform set up, ready to use whenever they wanted,^24 44^ whereas the ward setting required mindfulness not to disturb others and the need to be more emotionally ‘reserved’ when playing the games. The acute inpatient setting also had an impact on feelings about support from others. When training at home, support from family is widely recognised to facilitate use of rehabilitation technology^24 25 44^; however, our findings caution reliance on this support in the acute setting as time with family is far less, meaning time together is highly valued. Most participants required support initially until they became more familiar with device set up and use, whether that was physical support with using the platform or emotional support to encourage use. Future studies on the implementation of rehabilitation technology in the acute inpatient setting could further assess the additional workload this places on staff. In the community it could be explored whether this support needs to be provided in person or whether it could be provided remotely via telerehabilitation, given this can increase access to rehabilitation services.^45^

### Strengths and limitations

Acute and subacute stroke survivors were recruited, allowing the use of rehabilitation technology to be explored across the stroke pathway in both the hospital and home setting. The experience of participants in both the intervention and control groups was captured, with those from the intervention group representing a range of ages, severity of upper limb impairment and amount of device use. A limitation was that participants were recruited from a single hospital site and community team which could limit the transferability of findings. Additionally, the small number of individuals from the control group narrowed the range of insights offered. While undertaking the interviews immediately after completion of the intervention helped reduce the risk of recall bias, the views expressed were therefore immediate impressions rather than long-term reflections. Framework analysis was conducted in a rigorous and transparent manner by experienced qualitative researchers, with a clear audit trail. While the lead analyst was not involved in the delivery of the intervention or conducting of interviews, participants were interviewed by a member of the research team who was involved in the recruitment, device training and outcome measure collection. This may have impacted participant responses; however, the wide-ranging positive and negative views expressed suggest they felt open about sharing their experiences. The interviewer maintained a reflexive diary as a means of self-reflection throughout the process. While resource issues meant it was not possible to have an independent interviewer in this study, it is nevertheless recommended for future trials to help mitigate any potential interviewer bias. Member checking in this study was not undertaken due to concerns of overburdening the participants early in their stroke recovery when post-stroke fatigue is prevalent.

## Conclusion

Findings from this study demonstrate the positive impact that both rehabilitation technologies and involvement in rehabilitation research can have on individuals who have recently experienced a stroke. Acceptability of the Neurofenix platform was high, with participants enjoying the fun and competition of the games, and the ability to track their progress. All participants who completed the intervention period perceived the platform to have improved their upper limb function. Feedback gathered suggests that future development of the VR platform and other rehabilitation technologies should focus on personalisation with more varied and extensive game options to maintain adherence and engagement. Furthermore, to ensure potential participants are fully informed about study interventions, future trials should provide details of key device characteristics, for example, through provision of video examples. Insights offered from the acute setting also highlight the need for further work on improving device connectivity within hospitals and how individuals post stroke can best be supported to engage with technology-facilitated rehabilitation as they transition from hospital to home.

## Supplementary material

10.1136/bmjopen-2026-119145 online supplemental file 1

## Data Availability

No data are available.
