# Supplementary material for: Rehabilitation using virtual gaming for Hospital and hOMe-Based training for the Upper limb in acute and subacute Stroke (RHOMBUS II): a qualitative analysis of participants’ experience
Source: BMJ Open. 2026 Jul 2;16(7):e119145. doi: 10.1136/bmjopen-2026-119145 (PMC13331151; doi:10.1136/bmjopen-2026-119145)
Supplement: online supplemental file 1 [file bmjopen-16-7-s001.docx]

**
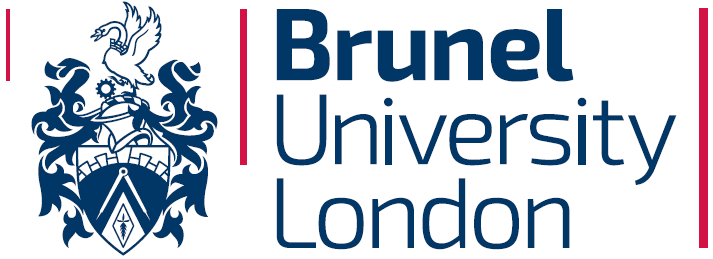
**


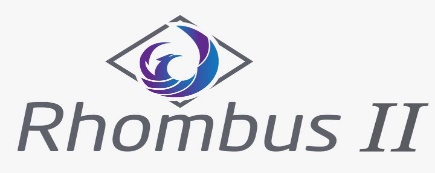


**RHOMBUS II Interview Topic Guide: intervention group**

**The content is indicative. Questions will not necessarily be asked in this order nor phrased in the way presented here.**

**Consent**

- Purposes of the interview:
  - to find out your views on the RHOMBUS II study, how it went for you, whether it made any difference to you, and how we can improve it.
  - To learn what you thought about taking part in the research study.
- So it is important to be as honest as you can so we know what works and what needs to change. You don’t have to answer any question, and can stop whenever you want, for instance if you feel tired – just say.
- I’ll record the interview so I can listen to you properly. What you say will remain confidential to the research team. Any quotes in reports of the study will be anonymised to protect your identity.
- Any time limitations or other issues with being interviewed now? It may last up to about an hour and a half.
- Obtain written consent.

**Overview of condition**

- Briefly, when you started the study what issues did you face following your stroke?
  - Prompts: UL use/sensation/fatigue/concentration/sensation/sitting balance
- How have these changed over the last 7 weeks?
- How do you feel about your recovery? (hopes, goals, fears)
- Where any other illnesses/conditions an issue for you at the time you had your stroke or in the last 7 weeks?

**Preparing to take part in RHOMBUS II**

- Why did you volunteer to take part in this study?
  - What were you hoping for from taking part in the RHOMBUS II study?
  - Prompts: goals, hopes for the content and process, how it would be run
- Did you know enough about what was involved in the study before you took part?
- Did you have any concerns about taking part? If so, what were these?

**Preparation for Intervention**

- At the start of the study your received training on the NeuroBall. How did you find the training?
  - Prompts:
    - What did you find helpful / unhelpful in the way they trained?
    - How well did they take account of your own needs/ circumstances (therapeutic alliance, fatigue)
    - At the end did you have enough knowledge to use it?
    - How confident did you feel to use it?
- What did you understand the purpose of the NeuroBall to be?
- You were also given some instruction guides/access to videos, what did you think of these?
  - Prompts:
    - Did it help / hinder your progress: how?
    - Was it easy to use?
    - Was the information helpful? In what way?
    - Was it missing any important information

**Questions regarding the NeuroBall^[[1]](#footnote-1)^ (bring device used as visual prompt)**

- How able were you to put on and use the device by yourself?
  - If no, what aspects did you need help with?
- What would make it easier to put it on?

**Using the NeuroBall**In your own words can you tell me about your experience of using the NeuroBall as part of this study?

*Prompt questions*

- - How did you feel using the Neuroball?
  - Was there anything in particular you liked / disliked about it?
  - Which activities did you like the most/least? Can you tell me why/what about it you liked/disliked?
  - How did you find the activities/games (challenging/interesting/boring)?
  - How much effort did you have to make to use the Neuroball?
  - Can you tell me about what you thought about the feedback it gave you (motivating/demotivating/distracting/humorous/insulting)
  - How did you find tracking your progress?
  - What did you think about additional features such as the leadersboard (possibly playing against someone else)?
  - Was it easy to pick which game you wanted to play and to use the menus? (Ease of navigating the games)
  - Did you suffer from any problems/adverse effects while using the NeuroBall (pain, motion sickness, headaches, falls, fear of falls, other concerns?)
    - If anything, what can we change to prevent these from happening?
  - What impact if any do you think the NeuroBall had with doing your arm and hand exercises? Prompt: motivation, adherence, time flies/length of time would exercise, burden
  - Do you think using the NeuroBall for 7 weeks had any effect on your arm? If so, can you describe the main effect you felt it had?
  - Was there anything that put you off using the Neuroball?
  - If the opportunity arose would you want to keep the Neuroball? Why?

**External Support**

- During the study did you receive support from anyone/where else with using the NeuroBall?
  Prompts:
  - Friends or family
  - Physiotherapist/private therapist
- And what support did they give you with using the NeuroBall?
- What did you think about the technical support offered? Was it adequate/lacking?

**General question inpatient and home use**

- How did it compare using the Neuroball at home compared to in the hospital? (prompt transition, oversight, fatigue, support, equipment, environment)

**Taking part in the research**

**Questions regarding the study:**

How did you find the assessment process – the questionnaires and physical assessments?

- - Meeting with the assessor
  - Completing the questionnaires
  - Performing the assessments
- How did you find communication with the research team (Vicky)
- Is there anything we could have done differently to make it a better experience for you?
- Would you be willing for us to contact you again in the future about this study or other studies related to stroke?
- Would you like to receive a report of this study
- Is there anything else you would like to say about the Neuroball or study?

**Conclusion**

**Thank them for their time.**

**
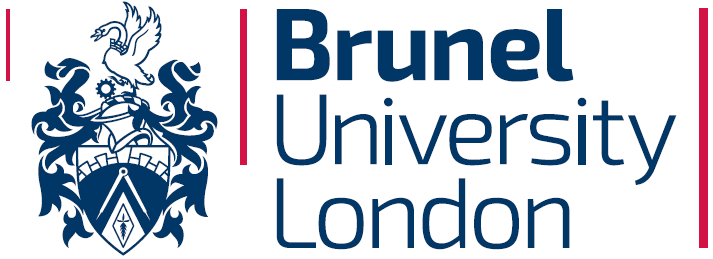
**
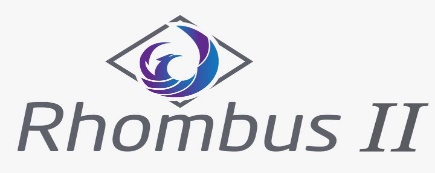


**Rhombus II Interview topic guide: control group**

Consent

- Purposes of the interview:
  - to find out whether the effects of your stroke have changed over recent months
  - To learn what you thought about taking part in the research study.
- Important to be as honest as you can so we know what works and what needs to change. You don’t have to answer any question, and can stop whenever you want, for instance if you feel tired – just say.
- I’ll record the interview so I can listen to you properly. What you say will remain confidential to the research team. Any quotes in reports of the study will be anonymised to protect your identity.
- Any time limitations or other issues with being interviewed now? It may last up to about half an hour.
- Obtain written consent.

Background

- Briefly, when you started the study what issues did you face following your stroke?
  - Prompts: UL use/sensation/fatigue/concentration/sensation/sitting balance
- How have these changed over the last 7 weeks?
- How do you feel about your recovery? (hopes, goals, fears)
- Where any other illnesses/conditions an issue for you at the time you had your stroke or in the last 7 weeks?

Experiences of being in the control group

- why did you volunteer for this study?
  - What did you hope might happen?
  - Prompts: outcomes, hopes for group allocation
- How did you feel about being put into the group that received usual care?
  - Did it affect your motivation to be in the study in any way?
- What and how much work have you been doing on your arm since being in the study?
  - With therapists
  - On your own – how
  - With others – classes, private therapists etc
- during the study period, have you received support from anywhere/anyone else in recovering from the effects of your stroke?
  - If so, please describe these
- Have you had any negative experiences that you think may have been a result of taking part in the programme or study?
  - Physical (accidents, injuries), psychological, social relationships, practical

Taking part in the research

- What did you think about the information you were given before joining the study?
  - Would you have liked more information about anything before deciding to take part?
- How did you find the assessment process
  - Meeting with the research assistant
  - Completing the questionnaires
- How did you find communication with the research team?
- Is there anything we could have done differently to make it a better experience for you?
- Do you want to receive a report of this study?
- Is there anything else you would like to say about taking part in this programme or study?

Conclusion

- Thanks for taking part

1. NeuroBall is the name of the hand controller for the NeuroPlatform. The term most familiar for the participant will be used [↑](#footnote-ref-1)
