# Supplementary material for: Rehabilitation using virtual gaming for Hospital and hOMe-Based training for the Upper limb in acute and subacute Stroke (RHOMBUS II): a qualitative analysis of participants’ experience
Source: BMJ Open. 2026 Jul 2;16(7):e119145. doi: 10.1136/bmjopen-2026-119145 (PMC13331151; doi:10.1136/bmjopen-2026-119145)
Supplement: Supplementary file 2 [file bmjopen-2026-119145.reviewer_comments.pdf]

PEER REVIEW HISTORY

BMJ Open publishes all reviews undertaken for accepted manuscripts. Reviewers are asked to complete a checklist review form and are provided with free text boxes to elaborate on their assessment. These free text comments are reproduced below.

ARTICLE DETAILS

Title (Provisional)

Rehabilitation using virtual gaming for Hospital and hOMe-Based training for the Upper limb in acute and subacute Stroke (RHOMBUS II): a qualitative analysis of participants' experience

Authors

Butcher, Tom; Warland, Alyson; Stewart, Victoria; Norris, Meriel; Aweid, Basaam; Samiyappan, Arul; Kal, Elmar; Ryan , Jennifer; Athanasiou, Dimitrios A.; Baker, Karen; Singla-Buxarrais, Guillem; Pound, Carole; Gowing, Francesca; Kilbride, Cherry

---

VERSION 1 - REVIEW

**Reviewer**	**1**
**Name**	**Wray, Faye**
**Affiliation**	**University of Leeds, Academic Unit for Ageing and Stroke Research**
**Date**	**01-May-2026**
**COI**	**None**

This manuscript reports a qualitative study exploring stroke survivors experiences of using a VR platform within a feasibility RCT. Understanding stroke survivors perspectives of such technology is highly important to ensure they are acceptable before larger scale evaluation. Overall, the manuscript is very well written and the methods are well reported. I have a few suggestions to further strengthen the manuscript:

1. The study aims

The aim is stated to be around exploring the acceptability of the VR platform and factors influencing the adoption of the intervention. However, it is clear from the findings/topic guide/inclusion of control group participants that the aims were also around exploring the acceptability of trial/data collection processes. The aims/abstract should be updated to clarify this.

2. Methods- Description of the intervention

I appreciate that the intervention is described in the protocol (thank-you for including the reference to this). However, I do think a brief description of the intervention is needed for the completeness of this paper. Without referring to the protocol/knowing the intervention, it is difficult to interpret the findings e.g. what weekly device use was expected? how long did the participants have access to the platform for? what support was provided to use the device? etc.

3. Methods/Findings

For interpretation of the findings it would be useful to know how many participants were approached to take part in the in-depth interviews and if anyone declined. This would be particularly useful context for interpretating the acceptability of allocation to the control group.

4. Results-Trial enrolment (theme one)

I am not really clear how this theme, particularly the findings around motivations for taking part in the study, relate to the study aims around exploring acceptability of the intervention or trial processes? Please could this be further clarified or the theme removed.

5. Results-Barriers and facilitators (theme three)

The opening line of the theme suggests that this is theme is related to environmental barriers and facilitators. However, the findings contained around pain/discomfort associated with using the device do not quite seem to fit with this. The theme name/description should be clarified to improve the flow within the theme.

6. Discussion- Cai et al. review

I am not clear how the findings from the Cai review around limited social support and feelings of abandonment relate to the findings of this study/how the findings from the study fill the research gaps identified by the review- please could this be further clarified.

7.Discussion-conclusion

An important finding around the trial processes was that some participants were not 100% clear what the intervention was/would involve. I think this should also be included in the conclusion as important learning from the study.

Reviewer 2

Name Lannin, Natasha

Affiliation Monash University Faculty of Medicine Nursing and Health Sciences, Neuroscience

Date 02-May-2026

COI None

The authors present a qualitative study conducted alongside a feasibility trial. The topic is valuable, and the study has the potential to contribute meaningfully to understanding participants' experiences of participating in complex rehabilitation trials. To ensure the manuscript aligns with expectations for qualitative research, several areas would benefit from further clarification and development (where able):

The study includes interviews with 11 intervention participants and 3 control participants. While this sample size is acceptable for qualitative work, the small number of control participants limits opportunities for comparison and may narrow the range of perspectives represented. It would be helpful for the authors to discuss this more explicitly as a study limitation. Relatedly, conducting the research within a single hospital and community team has implications for transferability, and acknowledging how this context shapes the diversity of experiences would enhance transparency.

As a single interviewer conducted all interviews, the authors could expand on how this may have influenced data collection and interpretation. Discussing how the interviewer's role within the research team might have shaped participant responses, and suggesting that future work consider multiple or independent interviewers, would strengthen the manuscript's reflexive account and address potential interviewer influence.

The use of Framework Analysis and the TFA is appropriate for the study aims. It may be useful to acknowledge that a primarily deductive approach can limit the emergence of unanticipated themes, and to note that future studies with the broader range of participants might additionally incorporate inductive coding alongside the TFA to capture a broader range of participant experiences.

Interviews were conducted shortly after the intervention. This timing may have emphasised immediate impressions rather than longer-term reflections, particularly regarding participants' perceptions of the intervention's value once outcomes become more apparent. A brief discussion of how interview timing may have shaped the findings is recommended.

There are also a number of edits required please to address the limitations of the paper:

Presentation of quotes and participant characteristics: The current use of pseudonyms may unintentionally imply gender, and the FMA-UE score alone provides limited information readily for the reader. A clearer and more neutral presentation (e.g., "P12- female, age 60, mild upper-limb impairment") would support reader understanding.

Reflexivity: The manuscript would benefit from a more explicit reflexive account. Outlining the research team's backgrounds, roles, and assumptions, and suggesting that future work incorporate more detailed reflexive practices (e.g., positionality statements, reflexive journaling), would enhance transparency and confirmability.

Discussion of perceived effectiveness: The paragraph describing perceived improvements in upper-limb function may be interpreted as evidence of intervention effectiveness, which is beyond the scope of qualitative findings. While perceived effects are appropriate within the

TFA theme, presenting them in the Discussion without reference to feasibility outcomes risks overinterpretation. I recommend removing this paragraph from the Discussion would avoid potential misinterpretation.

Member checking: As participant validation of transcripts or themes was not undertaken, it would be helpful to acknowledge this as a limitation. Given the use of a single interviewer who was part of the research team, recommending member checking or participant feedback in future studies would support confirmability and alignment with participants' intended meanings.

Limitations section: The current limitations section should be expanded to more comprehensively address methodological, sampling, analytic, and transferability considerations, as well as potential sources of bias.

VERSION 1 - AUTHOR RESPONSE

Reviewer: 1
Dr. Faye Wray, University of Leeds

Comments to the Author:
This manuscript reports a qualitative study exploring stroke survivors experiences of using a VR platform within a feasibility RCT. Understanding stroke survivors perspectives of such technology is highly important to ensure they are acceptable before larger scale evaluation. Overall, the manuscript is very well written and the methods are well reported. I have a few suggestions to further strengthen the manuscript:

Thank you for your kind words on the manuscript and helpful suggestions Dr Wray, please see below how we have amended the manuscript in response to your comments.

1. The study aims
The aim is stated to be around exploring the acceptability of the VR platform and factors influencing the adoption of the intervention. However, it is clear from the findings/topic guide/inclusion of control group participants that the aims were also around exploring the acceptability of trial/data collection processes. The aims/abstract should be updated to clarify this.
Within the abstract we have amended the aims by adding "and data collection processes" to the aim "to report acceptability of trial processes" in line 92. Within the main body we have also amended lines 193-194 to now say "A further aim was to explore the acceptability of trial processes including feelings about allocation to the control group".

2. Methods- Description of the intervention
I appreciate that the intervention is described in the protocol (thank-you for including the reference to this). However, I do think a brief description of the intervention is needed for the completeness of this paper. Without referring to the protocol/knowing the intervention, it is difficult to interpret the findings e.g. what weekly device use was expected? how long did the participants have access to the platform for? what support was provided to use the device? etc.
This is a good point, we have now added additional detail about the intervention, suggested

amount of device us and support provision in lines 208-216.

3. Methods/Findings
For interpretation of the findings it would be useful to know how many participants were approached to take part in the in-depth interviews and if anyone declined. This would be particularly useful context for interpretating the acceptability of allocation to the control group.
We have added in detail that three participants from the intervention group and one from the control group were invited but declined to take part in the interviews, see lines 269-270.

4. Results-Trial enrolment (theme one)
I am not really clear how this theme, particularly the findings around motivations for taking part in the study, relate to the study aims around exploring acceptability of the intervention or trial processes? Please could this be further clarified or the theme removed.
We have added an opening sentence now to theme one in lines 300-302 which hopefully clarifies how this theme relates to study recruitment processes and helps to inform future studies given this is a feasibility RCT.

5. Results-Barriers and facilitators (theme three)
The opening line of the theme suggests that this is theme is related to environmental barriers and facilitators. However, the findings contained around pain/discomfort associated with using the device do not quite seem to fit with this. The theme name/description should be clarified to improve the flow within the theme.
We have clarified that environmental barriers and facilitators is a sub-theme within the main theme and then have adjusted the start of the paragraphs on donning the NeuroBall and pain/discomfort to more clearly identify how they align with the themes as potential barriers. See lines 407, 420 and 430 for changes.

6. Discussion- Cai et al. review
I am not clear how the findings from the Cai review around limited social support and feelings of abandonment relate to the findings of this study/how the findings from the study fill the research gaps identified by the review- please could this be further clarified.
On reviewing this we completely understand this point. We have amended this to now more clearly state that the findings help to address the gap identified Cai et al that there is a need to explore how VR stroke rehabilitation can be used to create a continuum of care as stroke survivors transition from the hospital to home and have removed the sentence around addressing unmet needs. See lines 585-589.

7.Discussion-conclusion
An important finding around the trial processes was that some participants were not 100% clear what the intervention was/would involve. I think this should also be included in the conclusion as important learning from the study.
We have added a sentence to the conclusion to highlight this point in lines 689-691.

Reviewer: 2
Prof. Natasha Lannin, Monash University Faculty of Medicine Nursing and Health Sciences

Comments to the Author:

The authors present a qualitative study conducted alongside a feasibility trial. The topic is valuable, and the study has the potential to contribute meaningfully to understanding participants' experiences of participating in complex rehabilitation trials. To ensure the manuscript aligns with expectations for qualitative research, several areas would benefit from further clarification and development (where able):

Thank you for your review of our paper and helpful comments Professor Lannin, please see detail below on how we have tried to act on your suggestions.

The study includes interviews with 11 intervention participants and 3 control participants. While this sample size is acceptable for qualitative work, the small number of control participants limits opportunities for comparison and may narrow the range of perspectives represented. It would be helpful for the authors to discuss this more explicitly as a study limitation. Relatedly, conducting the research within a single hospital and community team has implications for transferability, and acknowledging how this context shapes the diversity of experiences would enhance transparency.
We have added a further limitation to the "strengths and limitations of the study" section which addresses this point in lines 141-142, and have also added it to the limitations section in the main body of the text, lines 663-665.

As a single interviewer conducted all interviews, the authors could expand on how this may have influenced data collection and interpretation. Discussing how the interviewer's role within the research team might have shaped participant responses, and suggesting that future work consider multiple or independent interviewers, would strengthen the manuscript's reflexive account and address potential interviewer influence.
We have now added some further detail on how the participants interacted with the interviewer during the trial and a recommendation for future trials to have an independent interviewer if possible. See lines 670-672 and 675-676.

The use of Framework Analysis and the TFA is appropriate for the study aims. It may be useful to acknowledge that a primarily deductive approach can limit the emergence of unanticipated themes, and to note that future studies with the broader range of participants might additionally incorporate inductive coding alongside the TFA to capture a broader range of participant experiences.
Apologies for any lack of clarity, however a hybrid deductive and inductive approach was taken to ensure unexpected data was captured, this is described in lines 248-251 "Transcripts were analysed using the Framework Method, which allowed for a hybrid deductive and inductive approach to be taken [31]. Deductive analysis was conducted using the pre-existing constructs of the TFA (table 1), however, to ensure all relevant and unexpected data was captured, inductive open-coding was used in parallel"

Interviews were conducted shortly after the intervention. This timing may have emphasised immediate impressions rather than longer-term reflections, particularly regarding participants' perceptions of the intervention's value once outcomes become more apparent. A brief discussion of how interview timing may have shaped the findings is recommended.
A point has now been made in the strengths and limitations section of the main text to suggest a strength in the interviewing timing of reducing recall bias, but that is also means views expressed are not long term reflections, see lines 665-668.

There are also a number of edits required please to address the limitations of the paper:

Presentation of quotes and participant characteristics: The current use of pseudonyms may unintentionally imply gender, and the FMA-UE score alone provides limited information readily for the reader. A clearer and more neutral presentation (e.g., “P12- female, age 60, mild upper-limb impairment”) would support reader understanding.
In response to your suggestion we have decided to remove the pseudonyms from the manuscript so as not to unintentionally imply gender (or ethnicity). We have now reported gender within table 2 and have amended the participant characteristics in the quotes. In response to the editors comment to not report exact ages we have also removed the age from the participant characteristics at the end of the quote.

Reflexivity: The manuscript would benefit from a more explicit reflexive account. Outlining the research team’s backgrounds, roles, and assumptions, and suggesting that future work incorporate more detailed reflexive practices (e.g., positionality statements, reflexive journaling), would enhance transparency and confirmability.
Additional detail has been added to the methods section to provide a more detailed reflexive account, including in lines 230-233 where the background and assumptions of the interviewer (VS) are detailed, and also the lead analysis background and role in lines 251-253.

Discussion of perceived effectiveness: The paragraph describing perceived improvements in upper-limb function may be interpreted as evidence of intervention effectiveness, which is beyond the scope of qualitative findings. While perceived effects are appropriate within the TFA theme, presenting them in the Discussion without reference to feasibility outcomes risks overinterpretation. I recommend removing this paragraph from the Discussion would avoid potential misinterpretation.
We would still like to include this paragraph within the discussion if possible, but have added in “In keeping with feasibility studies, this trial was not designed to examine or claim effectiveness…” in lines 626-627 to make it more clear that these are patient reported findings, not efficacy findings.

Member checking: As participant validation of transcripts or themes was not undertaken, it would be helpful to acknowledge this as a limitation. Given the use of a single interviewer who was part of the research team, recommending member checking or participant feedback in future studies would support confirmability and alignment with participants’ intended meanings.
This has been added to the strength and limitations section in lines 677-679.

Limitations section: The current limitations section should be expanded to more comprehensively address methodological, sampling, analytic, and transferability considerations, as well as potential sources of bias.
Hopefully our response to both yours and Dr Wray’s comments has now improved the limitations section of the paper.

VERSION 2 - REVIEW

**Reviewer**	**1**
**Name**	**Wray, Faye**
**Affiliation**	**University of Leeds, Academic Unit for Ageing and Stroke Research**
**Date**	**17-Jun-2026**
**COI**	

Thank-you for taking time to address my comments. I am satisfied with your revisions and am happy to recommend the manuscript is accepted.
